# Supplementary material for: Neuronal ensemble-specific DNA methylation strengthens engram stability
Source: Nat Commun. 2020 Jan 31;11:639. doi: 10.1038/s41467-020-14498-4 (PMC6994722; doi:10.1038/s41467-020-14498-4)
Supplement: Supplementary file 1 — Supplementary Information [file 41467_2020_14498_MOESM1_ESM.pdf]

Supplementary information

Neuronal ensemble-specific DNA methylation strengthens engram stability.

by Gulmez Karaca *et al*.

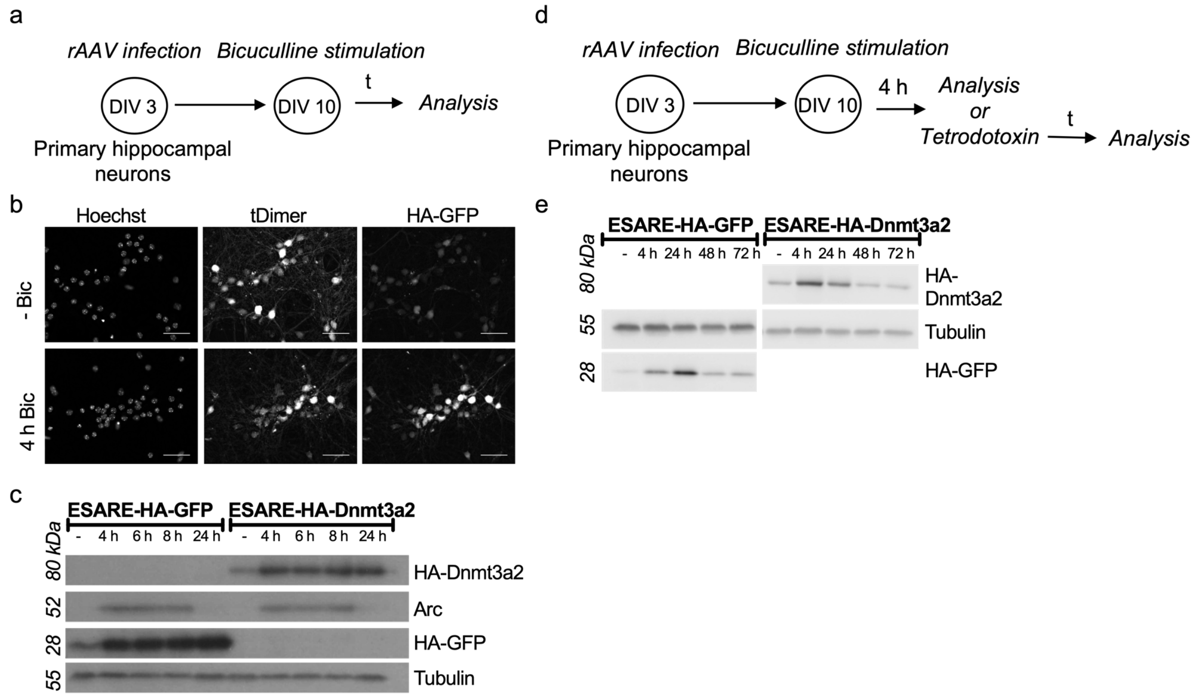

Supplementary Figure 1. Characterization of E-SARE-dependent gene expression in primary hippocampal cultures. (a) Experimental scheme used to characterize E-SARE dependent gene expression in primary hippocampal neurons. t indicates the duration of bicuculline stimulation. (b) Representative images showing the HA-GFP signal in primary hippocampal cultures after 4 h of bicuculline stimulation. Scale bar represents 50 μm. (c) Western blot analysis of HA-Dnmt3a2, HA-GFP and Arc expression in primary hippocampal cultures in baseline conditions or treated with bicuculline as indicated. Cultures were infected with E-SARE-HA-Dnmt3a2 or E-SARE-HA-GFP viruses. Tubulin served as a loading control (n = 4 independent culture preparations). (d) Experimental scheme used to determine the permanence of E-SARE-driven proteins in primary hippocampal neurons. After 4 h of bicuculline stimulation, neuronal activity was diminished by the application of tetrodotoxin. t indicates the time interval until protein harvesting. (e) Western blot analysis of HA-Dnmt3a2 and HA-GFP expression in primary hippocampal cultures in baseline conditions or treated with a pulse of bicuculline as indicated (note that the time points indicate the total time from the onset of bicuculline administration until protein harvesting) (n = 4 independent culture preparations). Cultures were infected with E-SARE-HA-Dnmt3a2 or E-SARE-HA-GFP viruses.

Tubulin served as a loading control. DIV: Day *in vitro*, Bic: Bicuculline, rAAVs: recombinant adeno-associated viruses. Source data are provided as a Source Data file.

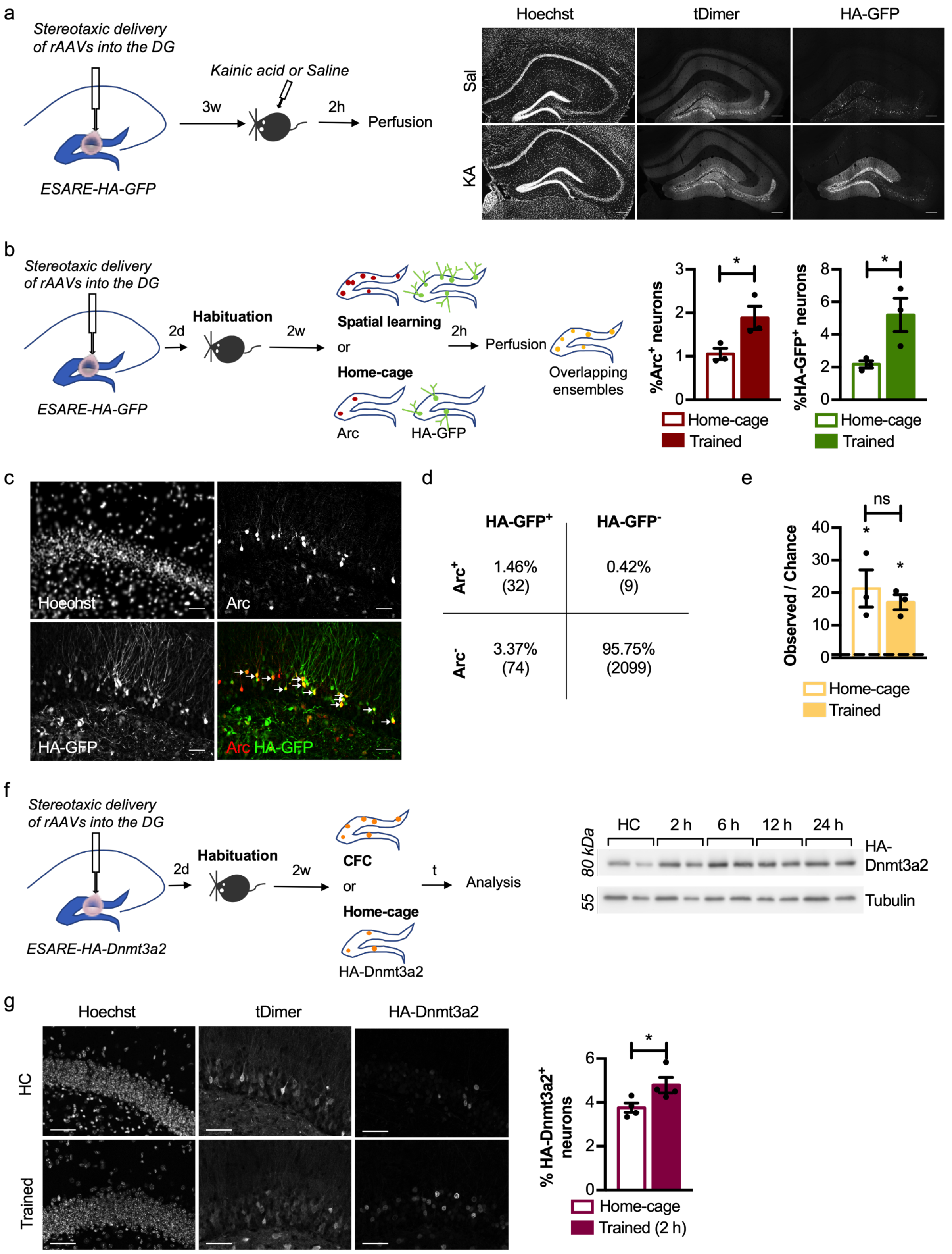

Supplementary Figure 2. Characterization of E-SARE-dependent gene expression in the mouse dentate gyrus. (a) Experimental scheme used to assess the effect of kainic acid administration on E-SARE dependent gene expression in the mouse hippocampus. Representative images showing the HA-GFP signal. Scale bar represents 200 μm. (b)

Behavioral scheme used to characterize the effect of spatial learning on E-SARE dependent gene expression in the mouse DG. Percentage of Arc^+ (Home-cage vs Trained: n = 3, t (4) = 2.804, p = 0.0486 by unpaired t-test) and $HA\text{-}GFP^+$ neurons (Home-cage vs Trained: n = 3, t (4) = 2.889, p = 0.0446 by unpaired t-test) in the DG of mice injected with E-SARE-HA-GFP. 2-3 brain slices per mouse were analyzed. (c) Representative images showing the immunohistochemical analysis of the overlap between Arc and HA-GFP signals in the DG of mice injected with E-SARE-HA-GFP. White arrows highlight the overlapping neurons. Scale bar represents 50 μm. (d) Quantification of the proportion and number of HA-GFP and / or Arc expressing neurons in the mouse DG. Data was obtained from three mice (2-3 brain slices per mouse). (e) Observed over chance overlap between Arc and HA-GFP expressions in the DG of the mice injected with E-SARE-HA-GFP (Observed overlap vs chance overlap: Home-cage: n = 3, t (2) = 5.378, p = 0.0329 by paired t-test; Trained: n = 3, t (2) = 5.987, p = 0.0268 by paired t-test; Overlap / Chance Home-cage (n = 3) vs Trained (n = 3): t (4) = 0.6905, p = 0.5278 by unpaired t-test). (f) Experimental scheme used to determine the effect of contextual fear conditioning on HA-Dnmt3a2 expression in the mouse DG (left). Western blot analysis of HA-Dnmt3a2 expression after contextual fear learning (right). Tubulin served as a loading control. t indicates the duration until the analysis. Two biological replicates were loaded in consecutive lanes for each time point. (g) Representative images showing the immunohistochemical analysis of HA-Dnmt3a2 and tDimer expression in the mouse DG. Scale bar represents 50 μm. Percentage of $HA\text{-}Dnmt3a2^+$ neurons (Home-cage vs Trained: n = 4, t (6) = 2.512, p = 0.0458 by unpaired t-test) in the DG of mice injected with E-SARE-HA-Dnmt3a2. 2-3 brain slices per mouse were analyzed. DIV: Day *in vitro*, Bic: Bicuculline, KA: Kainic acid, Sal: Saline, w: week, DG: Dentate gyrus of the hippocampus, CFC: contextual fear conditioning, rAAVs: recombinant adeno-associated viruses. *$p<0.05$; ns: not significant by the respective statistical test. Error bars represent s.e.m. Source data are provided as a Source Data file.

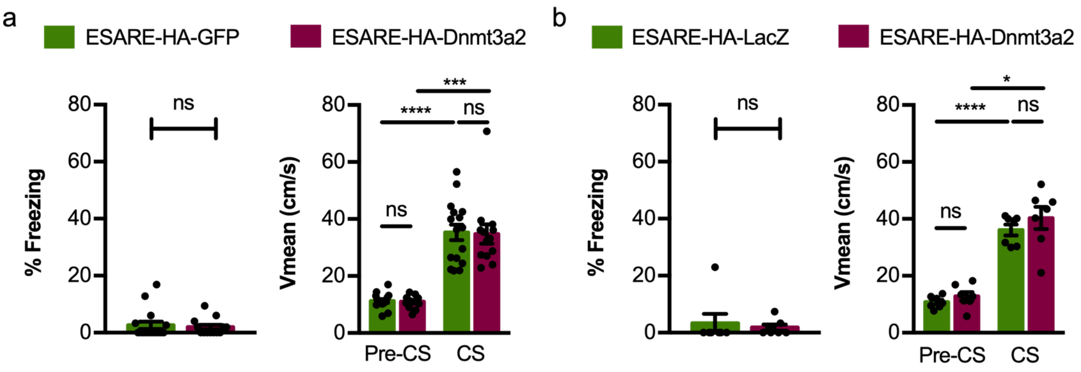

Supplementary Figure 3. Overall activity of mice injected with E-SARE-Control or E-SARE-HA-Dnmt3a2 prior and during shock administration. (a) Percentage of basal freezing rates and mean velocities during contextual-fear training of the mice injected with E-SARE-HA-GFP or E-SARE-HA-Dnmt3a2 and tested in the specific context (%Freezing: HA-GFP (n = 16) vs HA-Dnmt3a2 (n = 13), U = 96, p = 0.6994 by Mann-Whitney test) (Vmean: Pre-CS HA-GFP (n = 16) vs HA-Dnmt3a2 (n = 13), t (27) = 0.2738, p = 0.7863 by unpaired t-test; CS HA-GFP (n = 16) vs HA-Dnmt3a2 (n = 13), U = 93, p = 0.6501 by Mann-Whitney test; HA-GFP Pre-CS vs CS n = 16, t (15) = 8.891, p <0.0001 by paired t-test; HA-Dnmt3a2 Pre-CS vs CS n = 13, W = 91, p = 0.0002 by Wilcoxon test). (b) Percentage of basal freezing rates and mean velocities during contextual-fear training of the mice injected with E-SARE-HA-LacZ or E-SARE-HA-Dnmt3a2 and tested in a novel context (%Freezing: HA-LacZ (n = 7) vs HA-Dnmt3a2 (n = 7), U = 19, p = 0.5594 by Mann-Whitney test) (Vmean: Pre-CS HA-LacZ (n = 7) vs HA-Dnmt3a2 (n = 7), t (12) = 1.16, p = 0.2688 by unpaired t-test; CS HA-LacZ (n = 7) vs HA-Dnmt3a2 (n = 7), U = 12, p = 0.1282 by Mann-Whitney test; HA-LacZ Pre-CS vs CS n = 7, t (6) = 12.93, p <0.0001 by paired t-test; HA-Dnmt3a2 Pre-CS vs CS n = 7, W = 28, p = 0.0156 by Wilcoxon test). CS: Conditioned stimulus. *p<0.05; ***p<0.001; ****p<0.0001; ns: not significant by the respective statistical test. Error bars represent s.e.m. Source data are provided as a Source Data file.

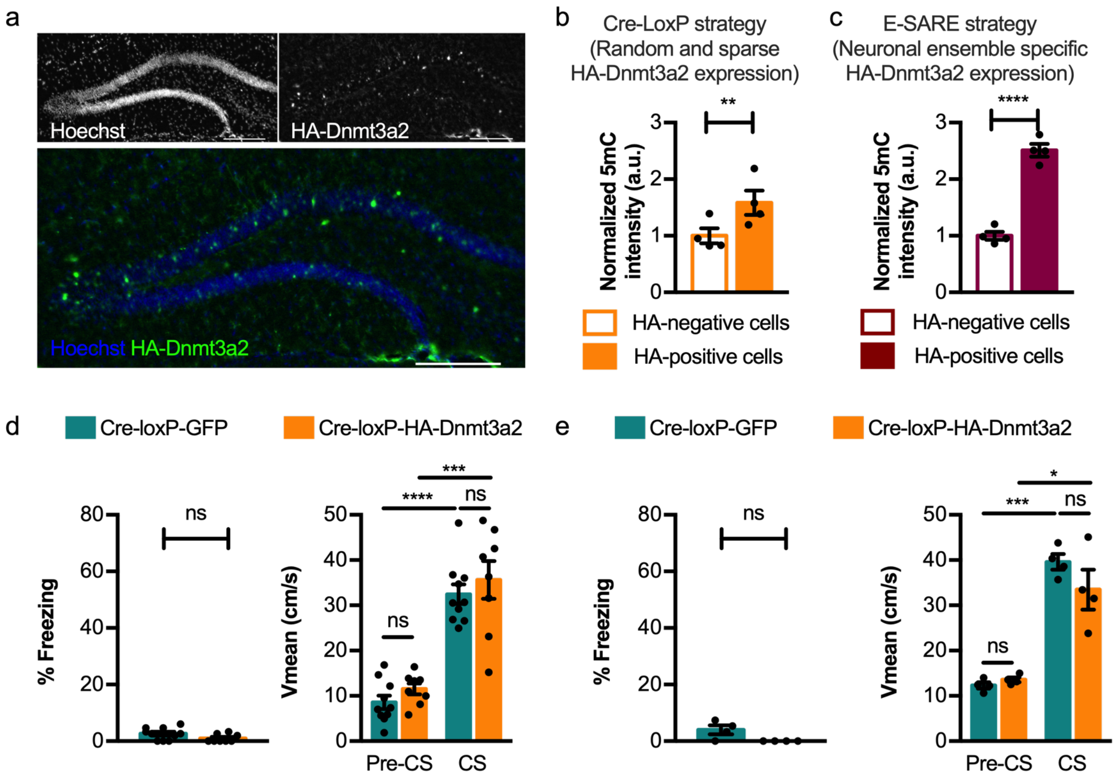

Supplementary Figure 4. Characterization of Cre-loxP-rAAV expression in the DG and the overall activities during contextual fear conditioning of the mice injected with Cre-LoxP-rAAVs. (a) Representative images of the immunohistochemical analysis of HA-Dnmt3a2 expression in the DG of the mice injected with Cre-loxP-rAAVs (5.682 % ± 0.6181 DG neurons expressed HA-Dnmt3a2, n = 4). Scale bar represents 200 μm. (b) Normalized mean intensity of 5mC signal in HA-positive and HA-negative neurons in the mouse DG expressing Cre-loxP-HA-Dnmt3a2 viruses (n = 4, t (3) = 6.483, p = 0.0074 by paired t-test). 2-3 brain slices per mouse were analyzed. (c) Normalized mean intensity of 5mC signal in HA-positive and HA-negative neurons in the mouse DG expressing E-SARE-HA-Dnmt3a2 viruses (n = 4, t (3) = 34.23, p < 0.0001 by paired t-test). 2-3 brain slices per mouse were analyzed. (d) Percentage of basal freezing (GFP (n = 10) vs HA-Dnmt3a2 (n = 8), U = 22.5, p = 0.1189 by Mann-Whitney test) and mean velocities during the training session of contextual-fear conditioning of the mice injected with Cre-LoxP-GFP or Cre-LoxP-HA-Dnmt3a2 (Pre-CS GFP (n = 10) vs HA-Dnmt3a2 (n = 8), t (16) = 1.478, p = 0.1590 by unpaired t-test; CS GFP (n = 10) vs HA-Dnmt3a2 (n = 8), t (16) = 0.7174, p = 0.4851 by unpaired t-test; GFP Pre-CS vs CS n = 10, t (9) = 9.253, p

<0.0001 by paired t-test; HA-Dnmt3a2 Pre-CS vs CS n = 8, t (7) = 6.412, p = 0.0004 by paired t-test). (e) Percentage of basal freezing and mean velocities of Cre-LoxP-GFP or Cre-LoxP-HA-Dnmt3a2 experimental groups during contextual-fear conditioning training (%Freezing: GFP (n = 4) vs HA-Dnmt3a2 (n = 4), U = 2, p = 0.1429 by Mann-Whitney test) (Vmean: Pre-CS GFP (n = 4) vs HA-Dnmt3a2 (n = 4), t (6) = 1.405, p = 0.2096 by unpaired t-test; CS GFP (n = 4) vs HA-Dnmt3a2 (n = 4), t (6) = 1.305, p = 0.2398 by unpaired t-test; GFP Pre-CS vs CS n = 14, t (3) = 13.1, p = 0.0010 by paired t-test; HA-Dnmt3a2 Pre-CS vs CS n = 4, t (3) = 5.117, p = 0.0144 by paired t-test). CS: Conditioned stimulus, a.u.: arbitrary unit. *p<0.05; ***p<0.001; ****p<0.0001; ns: not significant by the respective statistical test. Error bars represent s.e.m. Source data are provided as a Source Data file.

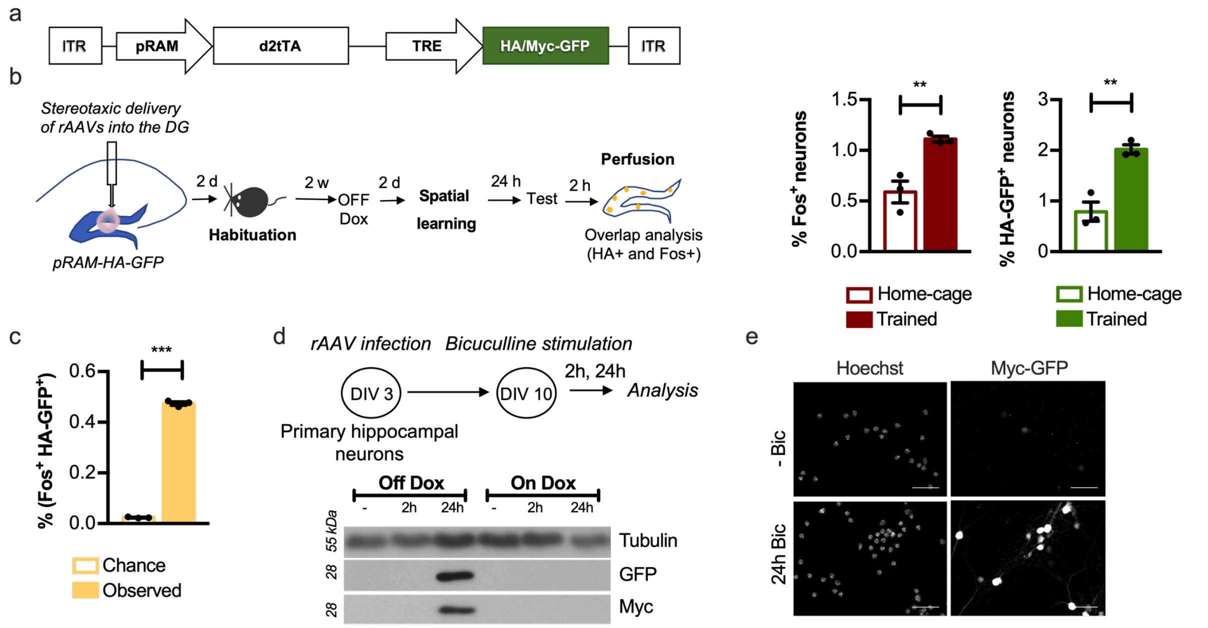

Supplementary Figure 5. Labeling of neuronal ensembles by the RAM promoter in primary hippocampal cultures and in the mice DG. (a) Schematic representation of the RAM-based viral vector. (b) Experimental scheme used to assess the effect of spatial learning on the expression of RAM-HA-GFP. Percentage of Fos$^+$ (Home-cage vs Trained: n = 3, t (4) = 4.732, p = 0.0091 by unpaired t-test) and HA-GFP$^+$ neurons (Home-cage vs Trained: n = 3, t (4) = 5.866, p = 0.0042 by unpaired t-test) in the DG of RAM-HA-GFP injected mice. (c) Observed overlap and chance overlap between Fos and GFP (Observed overlap vs chance overlap: n = 3, t (2) = 73.23, p = 0.0002 by paired t-test). (d) Experimental scheme used to assess the kinetics of RAM-myc-GFP expression upon neuronal activity in primary hippocampal cultures. Western blot analysis of myc-GFP expression in neuronal cultures infected with RAM-myc-GFP (n = 3 independent cell preparations). (e) Representative images showing the immunocytochemical analysis of pRAM-myc-GFP expression in primary hippocampal neurons upon synaptic activity in the absence of Doxycycline. Scale bar represents 50 μm. DIV: Day *in vitro*, Bic: Bicuculline, w: week, Dox: Doxycycline, DG: Dentate gyrus of the hippocampus, rAAVs: recombinant adeno-associated viruses. **p<0.01; ***p<0.001 by the respective statistical test. Error bars represent s.e.m. Source data are provided as a Source Data file.

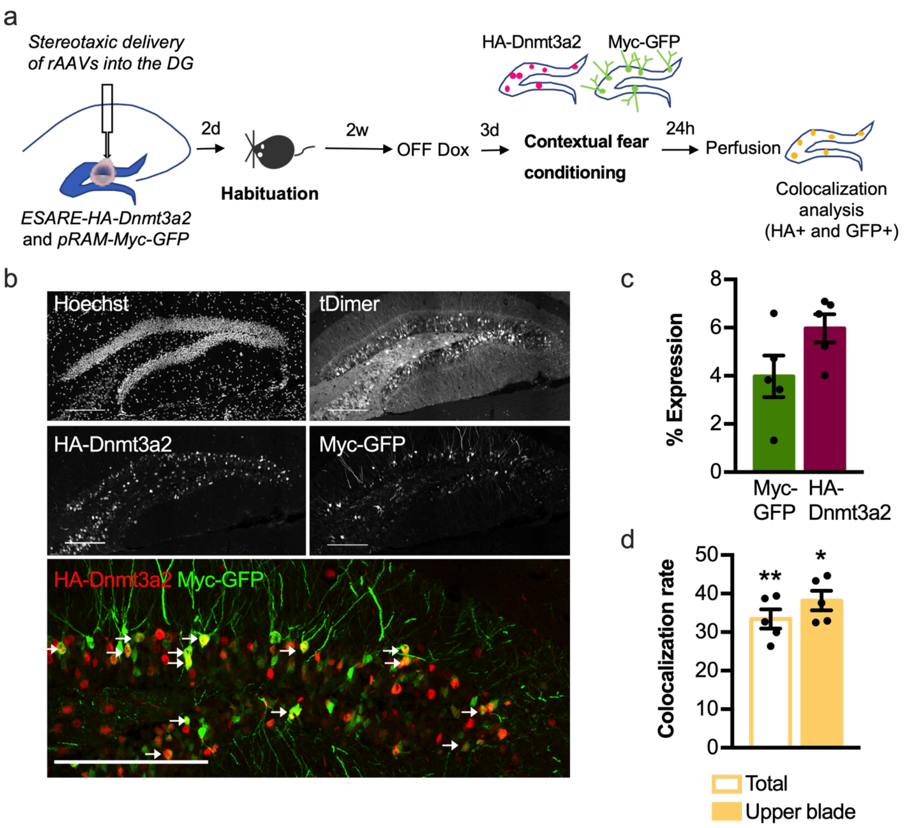

Supplementary Figure 6. RAM- and E-SARE-based neuronal ensemble tagging strategies significantly labelled the same population of neurons in the mouse DG. (a) Experimental scheme used to analyze the co-localization of RAM and E-SARE reporter proteins in the DG of the mice injected with E-SARE-HA-Dnmt3a2 and RAM-myc-GFP. (b) Representative images showing the immunohistochemical analysis of RAM-myc-GFP and E-SARE-HA-Dnmt3a2 in the DG. White arrows highlight the myc and HA co-expressing neurons. Scale bar represents 200 μm. (c) Percentage of neurons expressing myc-GFP or HA-Dnmt3a2 in the DG of the mice injected with E-SARE-HA-Dnmt3a2 and RAM-myc-GFP (n = 5). 2-3 brain slices per mouse were analyzed. (d) Colocalization rate of myc-GFP and HA-Dnmt3a2 signals in the mouse DG injected with RAM-myc-GFP and E-SARE-HA-Dnmt3a2 (Colocalization rate was calculated as following: $(GFP^{+}HA^{+}/GFP^{+})x100$; Total Observed overlap vs chance overlap: n = 5, t (4) = 4.666, p = 0.0095 by paired t-test; Upper blade Observed overlap vs chance overlap: n = 5, t (4) = 4.587, p = 0.0101 by paired t-test). 2-3 brain slices per mouse were analyzed. Dox: Doxycycline, w: week, DG: Dentate gyrus of the hippocampus, rAAVs: recombinant

adeno-associated viruses.*p<0.05; **p<0.01 by the respective statistical test. Error bars represent s.e.m. Source data are provided as a Source Data file.

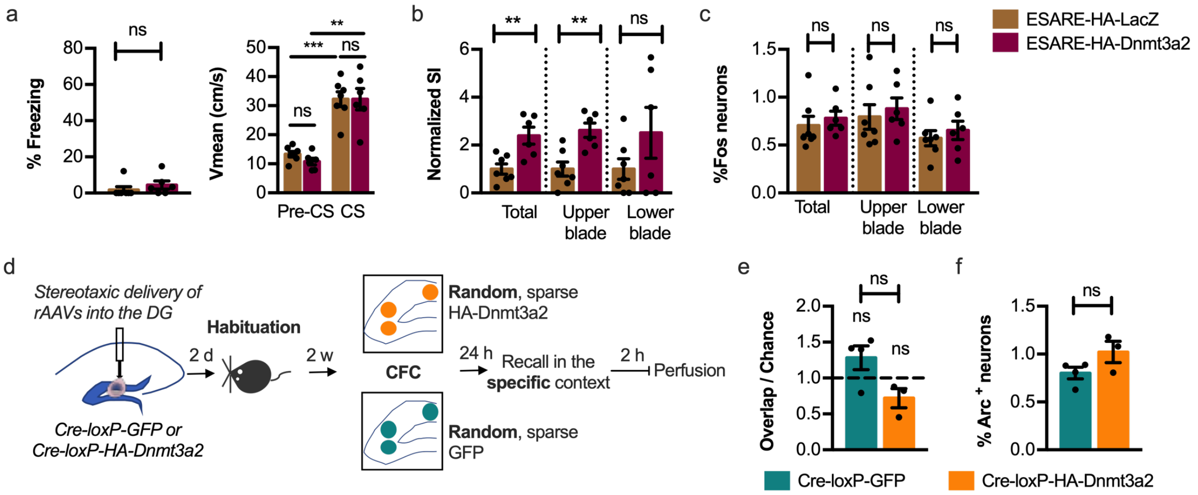

Supplementary Figure 7. Improved memory performances in the E-SARE-HA-Dnmt3a2 mice were associated with enhanced engram stability in the conditioning context. (a) Percentage of basal freezing rates prior to shock (HA-LacZ (n = 7) vs HA-Dnmt3a2 (n = 6), U = 11, p = 0.1189 by Mann-Whitney test) and overall activities during the contextual fear training of the mice injected with E-SARE-HA-LacZ or E-SARE-HA-Dnmt3a2 (Pre-CS HA-LacZ (n = 7) vs HA-Dnmt3a2 (n = 6), t (11) = 1.735, p = 0.1106 by unpaired t-test; CS HA-LacZ (n = 7) vs HA-Dnmt3a2 (n = 6), t (11) = 0.01142, p = 0.9911 by unpaired t-test; HA-LacZ Pre-CS vs CS n = 7, t (6) = 7.519, p = 0.0003 by paired t-test; HA-Dnmt3a2 Pre-CS vs CS n = 6, t (5) = 5.86, p = 0.0021 by paired t-test). (b) Normalized similarity indices in the DG of the mice injected with E-SARE-HA-LacZ or E-SARE-HA-Dnmt3a2 when the mice were tested in the fear-conditioned context (Total: HA-LacZ (n = 7) vs HA-Dnmt3a2 (n = 6), t (11) = 3.494, p = 0.0050 by unpaired t-test; Upper blade: HA-LacZ (n = 7) vs HA-Dnmt3a2 (n = 6), t (11) = 3.858, p = 0.0027 by unpaired t-test; Lower blade: HA-LacZ (n = 7) vs HA-Dnmt3a2 (n = 6), t (11) = 1.404, p = 0.1879 by unpaired t-test). 2-3 brain slices per mouse were analyzed. (c) Percentage of Fos^{+} DG neurons in the mice injected with E-SARE-HA-LacZ or E-SARE-HA-Dnmt3a2 after memory recall in the fear-conditioned context (Total: HA-LacZ (n = 7) vs HA-Dnmt3a2 (n = 6), U = 11, p = 0.1807 by Mann-Whitney test; Upper: HA-LacZ (n = 7) vs HA-Dnmt3a2 (n = 6), t (11) = 0.4933, p = 0.6315 by unpaired t-test; HA-LacZ (n = 7) vs HA-Dnmt3a2 (n = 6), t (11) = 0.6537, p = 0.5267 by unpaired t-test). (d) Experimental scheme used to assess the effect of random and sparse HA-Dnmt3a2 overexpression on Arc expression in the DG at memory

recall. (e) Observed over the chance overlap between Arc expression and GFP or HA-Dnmt3a2 expressions in the DG of the mice injected with Cre-loxP-GFP or Cre-loxP-HA-Dnmt3a2, respectively (Observed overlap vs chance overlap: GFP: n = 4, t (3) = 1.561, p = 0.2165 by paired t-test; HA-Dnmt3a2: n = 3, t (2) = 1.831 p = 0.2086 by paired t-test; Overlap / chance overlap GFP (n = 4) vs HA-Dnmt3a2 (n = 3), t (5) = 2.496, p = 0.0548 by unpaired t-test). 2-3 brain slices per mouse were analyzed. (f) Percentage of Arc^+ neurons in the DG of Cre-loxP-GFP or Cre-loxP-HA-Dnmt3a2 injected mice (GFP (n = 4) vs HA-Dnmt3a2 (n = 3), t (5) = 1.899, p = 0.1159 by unpaired t-test). 2-3 brain slices per mouse were analyzed. CFC: contextual fear conditioning, w: week, DG: Dentate gyrus of the hippocampus, SI: similarity index, rAAVs: recombinant adeno-associated viruses. *p<0.05; **p<0.01; ***p<0.001; ns: not significant by the respective statistical test. Error bars represent s.e.m. Source data are provided as a Source Data file.

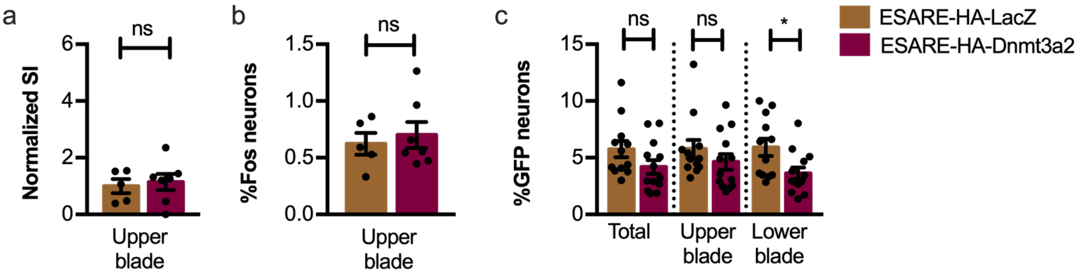

Supplementary Figure 8. Response of E-SARE-HA-Dnmt3a2 expressing neuronal ensembles to a novel context was not altered. (a) Normalized similarity indices in the DG of the mice injected with E-SARE-HA-LacZ or E-SARE-HA-Dnmt3a2 when the mice were tested in a novel context (HA-LacZ (n = 5) vs HA-Dnmt3a2 (n = 7), t (10) = 0.3704, p = 0.7188 by unpaired t-test). (b) Percentage of Fos^{+} neurons in the DG upper blade of E-SARE-HA-LacZ or E-SARE-HA-Dnmt3a2 injected mice (HA-LacZ (n = 5) vs HA-Dnmt3a2 (n = 7), t (10) = 0.4888, p = 0.6355 by unpaired t-test). 2-3 brain slices per mouse were analyzed. (c) Percentage of GFP^{+} neurons in the DG of the mice expressing E-SARE-HA-LacZ or E-SARE-HA-Dnmt3a2 (Total: HA-LacZ (n = 12) vs HA-Dnmt3a2 (n = 13), t (23) = 1.668, p = 0.1088 by unpaired t-test; Upper blade: HA-LacZ (n = 12) vs HA-Dnmt3a2 (n = 13), U = 54, p = 0.2051 by Mann-Whitney test; Lower blade HA-LacZ (n = 12) vs HA-Dnmt3a2 (n = 13), t (23) = 2.501, p = 0.0199 by unpaired t-test). 2-3 brain slices per mouse were analyzed. SI: similarity index. *p<0.05; ns: not significant by the respective statistical test. Error bars represent s.e.m. Source data are provided as a Source Data file.

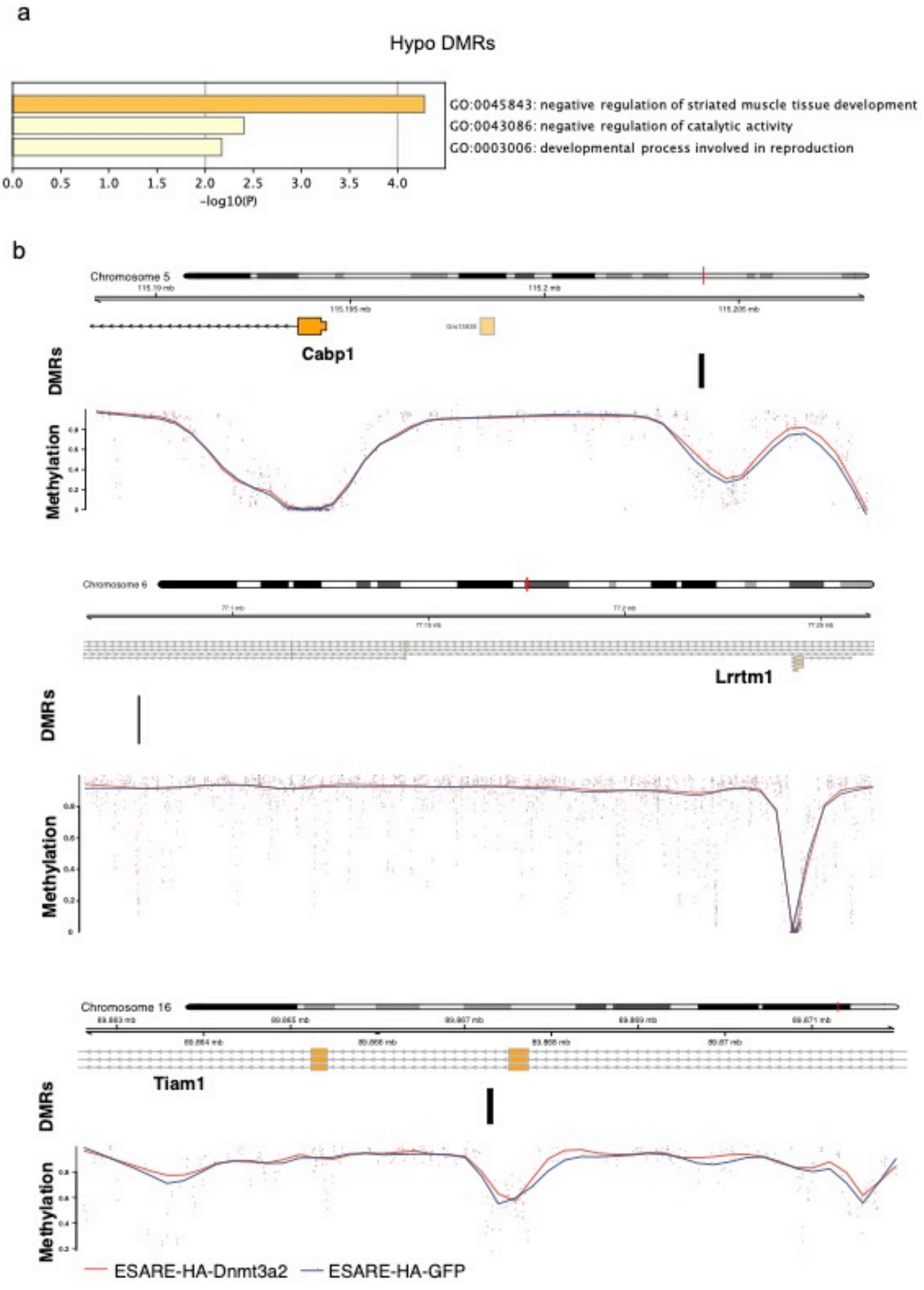

Supplementary Figure 9. DNA methylation changes driven by HA-Dnmt3a2 overexpression in primary hippocampal cultures. (a) Top GO (Gene ontology) categories found enriched using

the Metascape analysis of genes in the vicinity of hypo-DMRs. Shown are only categories with adjusted P>0.01 (hypergeometric test, Benjamini-Hochberg adjustment). (b) Examples of DMRs and associated loci in genome browser representation. DMR: differentially methylated regions, Hypo: hypomethylated.
