# Supplementary material for: Neuronal ensemble-specific DNA methylation strengthens engram stability
Source: Nat Commun. 2020 Jan 31;11:639. doi: 10.1038/s41467-020-14498-4 (PMC6994722; doi:10.1038/s41467-020-14498-4)
Supplement: Supplementary file 3 — Reporting Summary [file 41467_2020_14498_MOESM3_ESM.pdf]

nature research

Corresponding author(s): Ana MM Oliveira

Last updated by author(s): Dec 16, 2019

Reporting Summary

Nature Research wishes to improve the reproducibility of the work that we publish. This form provides structure for consistency and transparency in reporting. For further information on Nature Research policies, see Authors & Referees and the Editorial Policy Checklist .

Statistics

For all statistical analyses, confirm that the following items are present in the figure legend, table legend, main text, or Methods section.

n/a	Confirmed	
☐	☒	The exact sample size (n) for each experimental group/condition, given as a discrete number and unit of measurement
☐	☒	A statement on whether measurements were taken from distinct samples or whether the same sample was measured repeatedly
☐	☒	The statistical test(s) used AND whether they are one- or two-sided<br>*Only common tests should be described solely by name; describe more complex techniques in the Methods section.*
☐	☒	A description of all covariates tested
☐	☒	A description of any assumptions or corrections, such as tests of normality and adjustment for multiple comparisons
☐	☒	A full description of the statistical parameters including central tendency (e.g. means) or other basic estimates (e.g. regression coefficient) AND variation (e.g. standard deviation) or associated estimates of uncertainty (e.g. confidence intervals)
☐	☒	For null hypothesis testing, the test statistic (e.g. F, t, r) with confidence intervals, effect sizes, degrees of freedom and P value noted<br>*Give P values as exact values whenever suitable.*
☒	☐	For Bayesian analysis, information on the choice of priors and Markov chain Monte Carlo settings
☒	☐	For hierarchical and complex designs, identification of the appropriate level for tests and full reporting of outcomes
☐	☒	Estimates of effect sizes (e.g. Cohen's d, Pearson's r), indicating how they were calculated

Our web collection on statistics for biologists contains articles on many of the points above.

Software and code

Policy information about availability of computer code

Data collection	Equipment software of TSE systems fear conditioning setup, ChemiDoc imaging system, Nikon A1R confocal microscope, Illumina X-Ten.
Data analysis	Image J, GraphPad prism, R, methrix package, Bioconductor package DSS, ChIPseeker, Metascape. Data analysis detailed information is provided in the Methods section.

For manuscripts utilizing custom algorithms or software that are central to the research but not yet described in published literature, software must be made available to editors/reviewers. We strongly encourage code deposition in a community repository (e.g. GitHub). See the Nature Research guidelines for submitting code & software for further information.

Data

Policy information about availability of data

All manuscripts must include a data availability statement. This statement should provide the following information, where applicable:

- Accession codes, unique identifiers, or web links for publicly available datasets
- A list of figures that have associated raw data
- A description of any restrictions on data availability

The source data underlying Figs 1b-d, 2b,f,g, 3c-e, 4c-e and Supplementary Figs 1c,e, 2b,d-g, 3a,b, 4b-e, 5b-d, 6c,d, 7a-c,e,f, 8a-c are provided as a Source Data file. The WGBS data that support the findings of this study are available in European Nucleotide Archive (NEA) with the accession code PRJEB34831.

Field-specific reporting

Please select the one below that is the best fit for your research. If you are not sure, read the appropriate sections before making your selection.

☒ Life sciences ☐ Behavioural & social sciences ☐ Ecological, evolutionary & environmental sciences

Life sciences study design

All studies must disclose on these points even when the disclosure is negative.

Sample size	The sample size was determined based on similar experiments carried-out in the past.
Data exclusions	For behavioral experiments, we excluded data points from animals that exhibited insufficient viral spread or unintended viral spread in brain regions other than the target site. These criteria were established prior to data collection.
Replication	Each experiment represents 2 to 4 successfully replicated independent cohorts.
Randomization	Mice were randomly assigned to experimental groups. Every cohort included animals treated with control or manipulation viruses. The order of behavioral analysis was performed both randomly and blinded.
Blinding	For behavioral experiments the investigators were blind to group allocation during data collection and analysis. For in vitro experiments no blinding was performed since the outcome was dependent on software analysis and not manual scoring.

Reporting for specific materials, systems and methods

We require information from authors about some types of materials, experimental systems and methods used in many studies. Here, indicate whether each material, system or method listed is relevant to your study. If you are not sure if a list item applies to your research, read the appropriate section before selecting a response.

Materials & experimental systems

n/a	Involved in the study
☐	☒ Antibodies
☒	☐ Eukaryotic cell lines
☒	☐ Palaeontology
☐	☒ Animals and other organisms
☒	☐ Human research participants
☒	☐ Clinical data

Methods

n/a	Involved in the study
☒	☐ ChIP-seq
☒	☐ Flow cytometry
☒	☐ MRI-based neuroimaging

Antibodies

Antibodies used	For immunohistochemistry: HA-tag (Covance, MMS-101R (1:1000); Roche, #1867423 (1:100)), Arc (1:1000, Synaptic systems, 156003), Fos (1:1000, Cell Signaling, 2250), GFP (1:1000, Aves Labs, GFP-1020), 5mC (1:250, Active Motif, 39649). For immunocytochemistry: HA-tag (1:500, Santa Cruz, sc805), 5-mC (1:500, Calbiochem, NA81). For Western blotting: HA-tag (1:7500, Covance, MMS-101R), Arc (1:6000, Synaptic systems, 156003), Alpha-tubulin (1:400000, Sigma-Aldrich, T9026), hr-GFP (1:20000, Stratagene, 240142), myc-tag (1:500, Santa Cruz, sc-40).
Validation	All antibodies were purchased from commercial companies and have been validated by the companies.

Animals and other organisms

Policy information about studies involving animals; ARRIVE guidelines recommended for reporting animal research

Laboratory animals	We used male C57BL/6N mice (Charles River, Sulzfeld, Germany) that were 8-weeks old at the beginning of the experiments.
Wild animals	This study did not involve any wild animals.
Field-collected samples	No Field-collected samples were used in this study.
Ethics oversight	All procedures took place according to the German guidelines for the care and use of laboratory animals (Regierungspraesidium, Karlsruhe, Germany) and with the European Community Council Directive 86/609/EEC.

Note that full information on the approval of the study protocol must also be provided in the manuscript.
