# Supplementary material for: Neuronal ensemble-specific DNA methylation strengthens engram stability
Source: Nat Commun. 2020 Jan 31;11:639. doi: 10.1038/s41467-020-14498-4 (PMC6994722; doi:10.1038/s41467-020-14498-4)
Supplement: Supplementary file 4 — Description of Additional Supplementary Files [file 41467_2020_14498_MOESM4_ESM.pdf]

Description of Additional Supplementary Files

File name: Supplementary Data 1
Description: Differentially methylated regions (DMRs) in hippocampal neurons overexpressing Dnmt3a2.

File name: Supplementary Data 2
Description: Functional annotation of DMR-associated genes.
